# Chronic Abdominal Myoclonus Controlled with Prednisone Therapy: A Case Report

**DOI:** 10.7759/cureus.44178

**Published:** 2023-08-26

**Authors:** Priyanka Gurru, Kelash Bajaj, Manoher Lal

**Affiliations:** 1 Neurology, Ascension Seton Medical Center, Austin, USA; 2 Hematology and Oncology, Texas Tech University Health Science Center, Odessa, USA; 3 Neurology, Advanced Neuroscience Clinic, Midland, USA

**Keywords:** abdominal dyskinesia, neurology, prednisone, myoclonus, movement disorder

## Abstract

Abdominal myoclonus may manifest as involuntary, repetitive contractions of the abdominal wall due to a variety of neurologic pathologies. There are, however, limited cases reporting abdominal wall myoclonus without any clear neurologic etiologies. Here we present a case of a 72-year-old male with a history of rheumatoid arthritis, asthma, obstructive sleep apnea (OSA), and restless leg syndrome who presented with chronic, involuntary, repetitive contractions of his abdomen without any spinal or extremity involvement. His extensive neurologic and secondary systematic workup was negative, and he had a limited response to several different medication trials. The patient’s abdominal myoclonus, however, was better controlled with the administration of prednisone.

## Introduction

Abdominal wall myoclonus is a rare manifestation of certain diseases [1]. The clinical characteristics of this unique movement disorder are somewhat variable but usually consist of involuntary contractions of the abdominal wall muscles that cause abnormal jerking movements. They are differentiated from abdominal tremors due to their jerky quality with varying amplitudes, rather than low amplitude, rhythmic contractions. Etiologies of abdominal myoclonus have been reported to be medication side effects, vitamin B12 deficiency, abdominal surgery, childbirth, intramedullary thoracic cord tumors, osmotic demyelination, encephalitis, basal ganglia lesions, and epilepsy [1-6]. Epileptic myoclonic movements are generally present in a more stereotypic, episodic fashion. As mentioned, abnormal movements of the abdominal muscles may be caused by pathology in the brain or spinal cord leading to involuntary contractions of the abdominal wall musculature [7-9]. There are, however, a few reported cases of abdominal myoclonus in isolation of any known pathologic processes. Here we describe another unique presentation of a patient who presented to the neurology clinic with isolated abdominal myoclonus. 

## Case presentation

A 72-year-old right-handed man with a past medical history of rheumatoid arthritis, obstructive sleep apnea (OSP), asthma, and a remote appendectomy presented to the clinic with a three-year history of involuntary movements in his legs that spread in an ascending pattern to his abdomen. The patient reported that his symptoms began with numbness, tingling, and spasms in his left arm with associated cramps, stiffness, and locking of several muscles primarily on his left side. He had no deficits on the exam except for decreased sensation to vibration in his toes. His MRI C-spine only revealed mild cervical myelopathy and disc degeneration. Due to his excessive muscle stiffness, he was diagnosed with stiff-man syndrome and started on diazepam 2.5 mg BID and baclofen 10 mg TID with minimal improvement on follow-up. He continued the baclofen but stopped taking the diazepam. He was not seen in the neurology clinic for a year, during which he was diagnosed with restless leg syndrome after experiencing an uncomfortable, restless feeling in his legs while lying down to sleep, later followed by the emergence of small twitches. He was started on ropinirole 7 mg daily. When he returned to the clinic, he reported the jittery feeling in his legs had now escalated to involuntary, high frequency, and large amplitude jerking movements while falling asleep and seated in his recliner. These would temporarily resolve with walking. He noticed at this time small involuntary contractions of his abdominal muscles as well, which also significantly escalated within the next year to severe abdominal contractions, as seen in Video 1. Of note, the patient was being treated by his rheumatologist for his arthritis with 1 mg of prednisone TID throughout the same time period. He was not on DMARD therapy due to having a biopsy-positive coccidioides nodule in his lungs. While on prednisone, he would notice that increased doses of prednisone by a few milligrams would decrease the abdominal jerking. 

**Video 1:** Patient's abdominal myoclonus present at rest

One year into his condition, the patient had an episode of increased abdominal myoclonus that became severe enough to warrant a hospital admission. On admission, he was afebrile and hemodynamically stable. His neurologic exam was normal, except for the abdominal myoclonus and decreased bilateral pinprick sensation in his feet. His MRI brain and thoracic spine with and without contrast and CT scans were unrevealing. An EEG also showed no epileptic activity while his movements were occurring. His complete blood count (CBC), comprehensive metabolic panel (CMP), and uric acid (UA) were all normal as well. His erythrocyte sedimentation rate (ESR) and C-reactive protein (CRP) were also within range. Given that he was afebrile with a normal CBC, a lumbar puncture was not pursued. He was given lorazepam, diazepam, and valproate during admission with no response. He reported feeling a slight improvement on clonazepam, but there was no observable change in the abdominal myoclonus. He was then initiated on hydrocortisone 100 mg IV every eight hours, which helped decrease the myoclonic activity within a few days. Before discharge, he was placed on a prednisone taper; however, when tapered to 20 mg oral prednisone, his myoclonic activity worsened. An increase to 40 mg helped decrease the abnormal movements. Further outpatient workup revealed only a high vitamin B12 level and a low ferritin. He was negative for anti-glutamic acid decarboxylase (GAD) antibodies. He was also trialed on carbidopa-levodopa for Parkinson’s disease with no relief of symptoms. His care was escalated to a movement disorder specialist, who thought the etiology could be related to his restless leg syndrome and switched the patient to pramipexole, which he was not able to take because of side effects. He did not continue to follow up with the movement disorder specialist after his initial consult. 

The patient’s dose of ropinirole was then increased to 5 mg daily, and clonazepam 5 mg daily was added for symptomatic relief. He was most recently on a dose of prednisone 4 mg daily. He reported that these medications together provided relief from his restless legs and improved his abdominal movements, although they continue to persist. He did not want to pursue an LP at this time. 

## Discussion

We report a case of a patient with chronic, involuntary, jerking contractions of his abdomen, a rare type of myoclonus first discussed by Iliceto et al. [10]. These movements can be associated with various etiologies, including abdominal surgery, vaginal delivery, thoracic tumors, levodopa treatment, diaphragmatic flutter, tardive dyskinesia, basal ganglia lesions, and certain neuroleptics [1,7,11-13]. The exact pathophysiology of this condition remains unclear; however, it is often treated symptomatically after reaching a clinical diagnosis. Case reports have shown that diazepam and clonazepam have been effective in treating abdominal myoclonus by reducing the frequency and amplitude of abdominal contractions [1,8,14]. Along with those cases, there have also been a handful of cases of abdominal myoclonus with unclear etiology, similar to our patient whose imaging, labs, and medication history presented no clear etiology. The increase in the patient’s myoclonic symptoms after initiation of ropinirole was also investigated by discontinuing the drug, which led to the worsening of his restless leg syndrome with no improvement in his involuntary movements, decreasing the likelihood that the myoclonus was due to the medication. It has also been proposed that restless leg syndrome can occur and/or spread to the abdomen with associated propriospinal myoclonus, which is treated with pramipexole; however, our patient did not report feeling a restless abdomen and his symptoms were isolated to his abdominal wall musculature [15]. Nonetheless, an extreme case of restless leg syndrome may be considered as an etiology for the abnormal movements.

Most importantly, this patient’s interesting response to prednisone therapy also raises the question of a potential autoimmune or paraneoplastic cause of his myoclonus. With a history of rheumatoid arthritis and worsening of symptoms on decreased doses of prednisone, an autoimmune etiology may be considered. Autoimmune movement disorders have been seen to occur in isolation or in association with other diffuse autoimmune encephalitic illnesses [16]. Autoantibodies to LG1 and glutamic acid decarboxylase 65-kilodalton isoform (GAD65) have been implicated in myoclonic autoimmune conditions, such as stiff person syndrome, an initial consideration in our patient [16]. Although autoimmune movement disorders often present with other associated symptoms, our patient’s response to prednisone makes a potential autoimmune etiology a consideration. Finally, the exact effects of corticosteroids directly on the nervous system are not very well understood. The CNS has densely located corticosteroid receptors in the brain, and studies have shown that corticosteroids may alter brain excitability by altering cyclic nucleotide metabolism, interacting with neural membranes, altering ionic conductance, and potentially altering the activity of monoamine neurotransmitters [17]. These effects, however, have not been extensively investigated in human neurochemistry in relation to neurologic diseases. Therefore, prednisone therapy in individuals with neurologic disorders may be due to direct effects on the CNS itself, which may be true for our patient as well. With all this considered, prednisone may therefore be tried as a treatment for a rare abdominal myoclonus that presents with no clear etiology and is refractory to other treatments. 

## Conclusions

Abdominal myoclonus from an underdetermined etiology is a rare neurologic presentation. Patients have been previously treated with clonazepam and diazepam with limited relief. The response to prednisone treatment in our patient makes steroid therapy an option for those patients diagnosed with abdominal myoclonus.
